# Impact of the Circadian Rhythm and Seasonal Changes on the Outcome of Cardiovascular Interventions

**DOI:** 10.3390/jcm14082570

**Published:** 2025-04-09

**Authors:** Marc Licker, Christoph Ellenberger

**Affiliations:** 1 Department of Cardiovascular & Thoracic Anaesthesia and Critical Care, University Hospital of Martinique, F-97200 Fort de France, France; marc.licker@unige.ch; 2 Faculty of Medicine, University of Geneva, CH-1211 Geneva, Switzerland; 3 Department of Acute Medicine, University Hospital of Geneva, CH-1205 Geneva, Switzerland

**Keywords:** daytime variation, myocardial ischemia, chronobiology, chronotherapy, oxidative stress

## Abstract

The activities of living beings fluctuate according to seasonal changes and circadian rhythms. The interaction of organisms with their environment, notably weather conditions and night–day cycles, modulate homeostatic mechanisms and influence physiological responses in stressful situations. In humans, it is well established that cardiovascular events such as myocardial infarction, stroke and acute heart failure more frequently occur in winter than in summer season (non-tropical regions) and in the morning than in the evening. While the effects of cardiovascular medications vary during the day, the influence of circadian rhythms on the outcomes of invasive interventions is the subject of conflicting debates. This paper analyzes the impact of seasonal variability and circadian rhythms on physiological responses and the occurrence of complications in cardiac surgery and interventional cardiology.

## 1. Introduction

In the Bible, God’s creation of the Universe begins with the separation of light and darkness (Book of Genesis 1. 1–25). In Antiquity, the importance of life cycles was emphasized when Hippocrates reported the cyclical variations in pathological processes [1] while Sophocles, Virgil and Ovid constantly questioned sleep–wake cycles in their literary works [2].

From the beginning of the 19th century, the work of chronobiologists demonstrated that all living organisms were subjected to seasonal variations, as determined by the revolution of the earth around the sun, as well as to circadian variations (circa–around, diem–day) according to the “day–night” alternation determined by the rotation of the earth on its own axis [3]. In 2017, the Nobel Prize in Medicine was awarded to three chronobiologists, Jeffrey C. Hall, Michael Risbash and Michael W. Young, for their discoveries regarding the mechanisms of circadian rhythms and their impact on physiological processes, as well as the emergence and modulation of various diseases [4].

Nowadays, cardiovascular diseases, including myocardial infarction, heart failure, arrhythmias and stroke, remain the leading causes of mortality worldwide [5]. Interactions between the genetic fingerprint of individuals and extrinsic factors such as a sedentary lifestyle, poor socioeconomic conditions, diet and stress levels all contribute to the development of high blood pressure, diabetes mellitus, and renal failure associated with the formation of atherosclerotic plaques in the coronary, cerebral, renal and peripheral vessels [6]. Concerning environmental stress factors, variations in weather conditions and different levels of exposure to light are emerging as new cardiovascular risk factors.

## 2. Seasonal Variations

Epidemiological studies based on national death registers clearly demonstrate excess cardiovascular mortality in the northern and southern hemispheres in winter (+15 to 35%); this is linked to peaks in the incidence of myocardial infarctions, strokes, heart decompensation, as well as dissection of the thoracic and abdominal aorta [7,8,9,10]. Importantly, the increased risk of cardiovascular events in autumn/winter, compared to spring/summer, is largely correlated to variations in weather conditions (temperature, humidity level, atmospheric pressure, precipitation, winds), pollution peaks, as well as the duration of sunshine.

**Figure 1:**
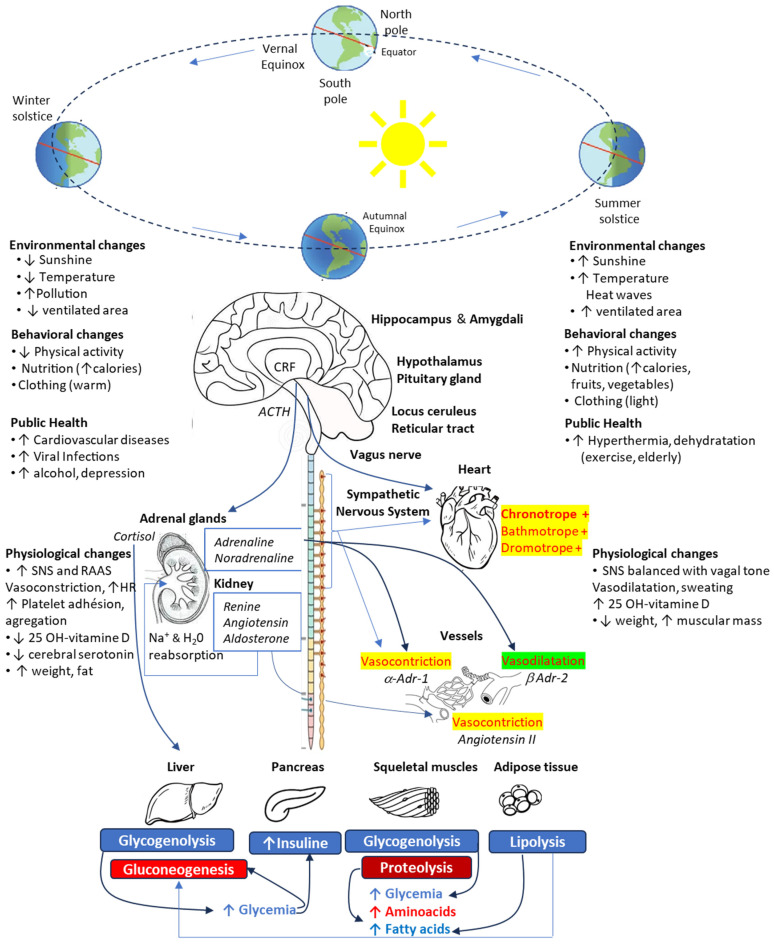
Impact of seasonal changes on the environment and the interactions with human behaviors and homeostatic mechanisms. ACTH, adrenocorticotropic hormone; CRH, corticotropin-releasing hormone; RAAS, renin–angiotensin–aldosterone system; SNS, sympathetic nervous system.

During cold exposure, the hypothalamus functions like a thermostat: it detects any temperature deviation from the equilibrium point of 37 °C and triggers the physiological responses aimed at reducing caloric losses (cutaneous vasoconstriction, piloerection) and producing heat by increasing the metabolism caused by muscular contractions due to shivering [11]. These thermoregulatory responses activate the hypothalamic–pituitary–adrenal axis, resulting in increased cortisol levels; this allows the mobilization of energy substrates while the stimulation of the sympathetic nervous and renin–angiotensin–aldosterone systems results in peripheral vasoconstriction and water/salt retention, as well as increases in heart rate, blood pressure and platelet aggregability (Figure 1).

In countries with temperate regions, the risk of cardiovascular events is increased during winter and autumn seasons, particularly among hypertensive, diabetic or obese subjects due to neurohumoral activation, causing an overload in cardiac work, a prothrombotic state, higher circulating cholesterol levels and weakening of immune defenses [12]. In the epithelial cells of the respiratory tract and the pulmonary parenchyma, microbes encounter more favorable conditions for their proliferation, especially when people confine themselves to poorly ventilated spaces, facilitating the inter-individual transmission of microorganisms [13]. On the other hand, low exposure to ultraviolet rays leads to reduced synthesis of 25 OH-vitamin D, which may contribute to hypertensive peaks, cardiac decompensation and coronary ischemic events [14].

The complexity of interactions between individuals and their environment cannot solely be reduced to dose–response effects triggered by the magnitude of changes in atmospheric conditions. The relationship between mortality and temperature corresponds graphically to a U-shaped curve, taking into account the deleterious effects of heat waves and extreme cold [15]. Noteworthy, temperature oscillations induce greater excess mortality in populations in tropical regions, which are little accustomed to low temperatures, than in continental regions where the population becomes more resistant to periodical cold exposure via homeostatic modifications, individual protective behaviors (clothing, diet, exercises) and collective logistical adjustments (insulation and heating of public buildings, commercial centers and private houses) [16].

## 3. Circadian Rhythms

### 3.1. Central and Peripheral Clocks

**Figure 2:**
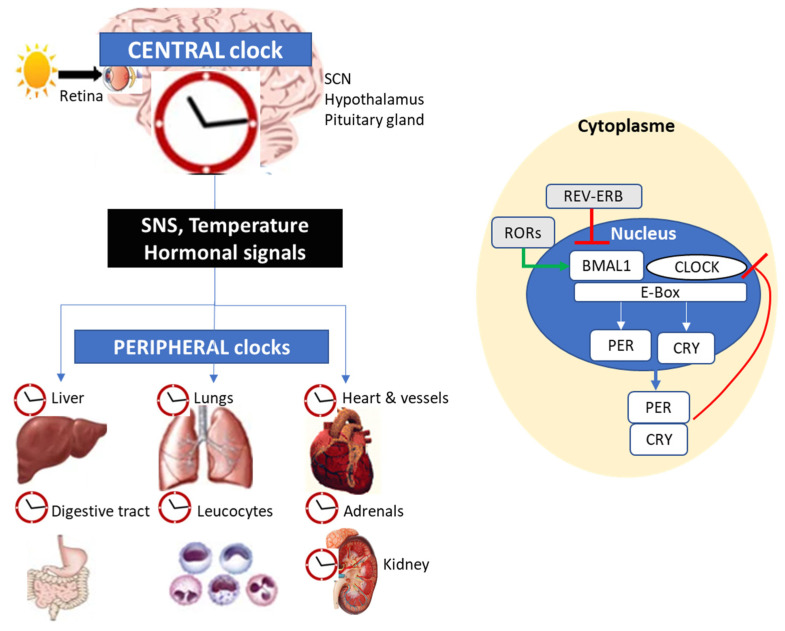
Physiological responses during circadian cycle. BMAL1, brain and muscle Arnt-like protein-1; CLOCK, circadian locomotor output cycle kaput; CRY, cryptochrome; LDL, low-density lipoprotein; E-Box, enhancer box (DNA motifs with the sequence CANNTG); PER, period; ROR, retinoid-related orphan receptor; SCN, supra-chiasmatic nucleus; SNS, sympathetic nervous system.

In mammals, the circadian rhythm of biological processes is tightly coupled to sleep–wake cycles by a central or master clock located at the level of the suprachiasmatic nuclei (SCN) (Figure 2) [17]. Up to 20% of the genome is under circadian regulation. Time synchronization in this central clock is determined by the light-induced stimulation of retinal ganglionic cells that transmit signals to the SCN and in turn entrain neurons in other brain areas, the hypothalamus and the pituitary gland. The pacemaker activity of the SCN directly or indirectly influences organ function and immunity by synchronizing the peripheral clocks present in many tissues (i.e., liver, kidney, heart vessels, adrenals, kidney and leucocytes); by the sympathetic nervous system and hormonal signaling pathways (corticotropin, cortisol, melatonin) [17].

At the molecular level in both central and peripheral clocks, a complex network of transcription–translation feedback loops regulates the rhythmic expression of clock-related genes (Figure 2). The heterodimeric interactions between two transcription factors, i.e., the brain and muscle Arnt-like protein 1 (ARNTL, also known as BMAL1) and the circadian locomotor output cycles kaput (CLOCK) represent central mechanisms of the circadian rhythm in mammals [18]. After heterodimerization in the cytoplasm, the BMAL1:CLOCK complex translocates into the nucleus, where it binds to canonical Enhancer Box (E-Box) sequences, promoting the transcription of period (PER1-3) and cryptochrome (CRY1-2); these exert a negative feedback on the transcription of the BMAL1:CLOCK complex [19]. Since CRY and PER concentrations decrease at night, their inhibitory activity is alleviated, allowing a new cycle of CLOCK-BMAL1 complex-mediated transcriptional activation.

The nuclear hormone receptors REV-ERBα and REV-ERBβ, as well as the retinoic acid orphan receptor (ROR), are other key regulators of the circadian clock, lipid and glucose metabolism, as well as cellular differentiation [18]. The REV-ERBs act as transcriptional repressors of the expression of BMAL1, whereas RORs positively regulate the expression of BMAL1 by binding to Retinoic acid receptor-related Orphan Receptor Element (RORE) elements in the BMAL1 gene promoter. Changes in the degradation speed of circadian proteins owing to post-translational phosphorylation and proteolysis result in synchronizing clock oscillation [17,18,19].

Central and peripheral biological clocks coordinate the function of body organs, with the aim of promoting efficient energy expenditure during periods of intense metabolic activity while allowing recovery phases to maintain homeostasis, facilitate healing processes and restore energy reserves. Thus, the morning release of cortisol associated with the stimulation of the sympathetic nervous system induces glycogenolysis and lipolysis to support the aerobic metabolism associated with physical and intellectual activities during the daytime period; meanwhile, neuro-humoral deactivation in the evening is coupled with the release of melatonin, which promotes anabolic energy-restoring activities, reduces wakefulness and initiates sleep [18].

### 3.2. Circadian Cardiovascular Rhythms and Pathological Processes

#### 3.2.1. Underlying Mechanisms

**Figure 3:**
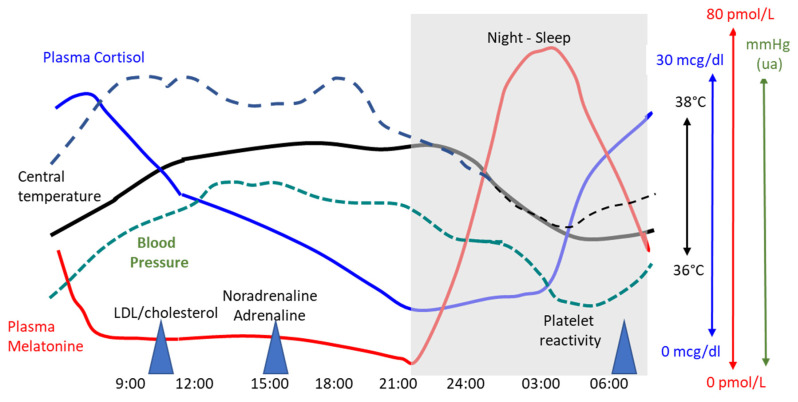
Circadian changes in central temperature, blood pressure, HR, heart rate, platelet reactivity, lipids and hormones (noradrenaline, adrenaline, melatonin). LDL, low-density lipoprotein.

Daily fluctuations in cardiovascular parameters are synchronized at the level of the SCN and peripheral clocks (Figure 3). Animal studies have revealed the presence of CLOCK, BMAL1, PERIOD1/2, and CRYPTOCHROMES in most cardiovascular tissues, which drive various output genes to control cellular protein synthesis and metabolic processes, modulate the capacity for cellular and mitochondrial self-destruction (necrosis, autophagy, apoptosis), and modulate the degree of oxidative and inflammatory stress [20,21]. These molecular mechanisms are also influenced by aging, physical activity, smoking behavior and eating habits. Importantly, during ischemia–reperfusion episodes, the severity of myocardial damage is influenced by circadian genes PER/CRY (negative during sleep) and core clock genes CLOCK/BAML1 (positive during sleep). Noteworthy, genetic polymorphisms within the core clock genes BMAL1 and CLOCK are associated with several cardiovascular metabolic diseases, namely hypertension, vascular atheromatosis, obesity and diabetes mellitus [22,23,24].

The response of cardiomyocytes and vascular tissues to physiological stimuli (e.g., insulin, catecholamines, fatty acids) and pathological stresses (e.g., ischemia-reperfusion, arterial hypertension) is modulated by peripheral clocks via transcription–translation loops involving growth factors (i.e., growth hormone, GH; insulin-growth factor, IGF) and metabolic biomarkers (substrates and enzymes of glycolysis and the Krebs cycle) [25]. Hence, the pattern of myocardial contractility and relaxation, intracellular energy processes, ECG waveforms, endothelial and smooth muscle reactivity, and electrical conduction all exhibit circadian variations.

#### 3.2.2. Blood Pressure Control

Under physiological conditions, blood pressure (BP) and heart rate exhibit a morning peak followed by a gradual drop and a nadir in the middle of the night (−10 to 15 beats/min, −10 to 20% blood pressure, respectively); variations in the activation of the autonomic nervous system, the endothelial release of nitric oxide (NO), platelet aggregability, and adhesiveness correspond to these changes. The dipping index is defined as the percentage decrease in the average nocturnal BP, compared with the mean daily BP. These circadian BP variations reflect the neuroendocrine modulation and its interactions with physical activity, emotional status, environmental parameters (e.g., temperature and humidity, oral intakes (e.g., nutrients, vasoactive xenobiotics, alcohol and caffeine), and sleep patterns. Subjects with an attenuated BP decline at night, that is, non-dippers (<10% decline) or individuals with nocturnal hypertension (riser profile) show an increased risk of adverse cardiovascular events as a result of increased vascular shear stress, causing the rupture of atherosclerotic plaques. Not surprisingly, myocardial infarcts and strokes are both more frequent and more serious between 3 a.m. and 12 p.m., rather than in the second part of the day, due to higher hemodynamic constraints, an accentuated prothrombotic state, and a relatively poor tolerance to ischemia–reperfusion episodes [26]. Furthermore, cardiovascular events are more likely to occur in the elderly, workers with irregular schedules (days/nights or travel with “jet lag”), and hospitalized patients in intensive care when the circadian rhythm is disrupted [27].

#### 3.2.3. Chronotherapy

As the occurrence of acute cardiovascular events shows time-dependent patterns, chronotherapy, i.e., time-tailored therapeutic approaches, may improve cardiovascular outcomes [28].

A systematic review of 25 studies revealed that circadian rhythmicity generates a pro-thrombotic state, as reflected by hypofibrinolysis and hypercoagulation in the morning hours [29]. The renin–angiotensin system (RAS) also follows circadian rhythmicity, with overactivity during the night hours [30]. Therefore, to prevent myocardial infarct and treat hypertension and heart failure, angiotensin-converting enzyme inhibitors (ACEI) and angiotensin blockers should preferably be taken in the evening to achieve their maximal effects in lowering peripheral resistance and blood volume at night and in the early morning [31]. Similarly, aspirin should also be prescribed at bedtime to reduce platelet reactivity and prevent the occurrence of ischemic heart disease [31,32]. In a meta-analysis comparing clinical trials where antihypertensive medications were given in the evening vs. morning, a 48% reduction in the relative risk of adverse CV events was reported in patients taking their medications in the evening compared to the morning [33]. In contrast with these findings, the TIME study including 21,104 patients in the UK failed to demonstrate any difference in the primary cardiovascular outcome (death or hospitalization for non-fatal myocardial infarct or stroke) in patients randomized to an evening vs. morning dosing of antihypertensives [34].

## 4. Natural Rhythms and Cardiovascular Interventions

### 4.1. Seasonal Variations

Seasonal fluctuations in acute cardiovascular events such as unstable angina, MI, heart failure and hypertensive crisis influence the number and severity of cardiovascular interventions performed at different periods during the year [35,36]. From a systematic review including 128,101 patients (28 trials), interventions for dissections of the thoracic aorta and congenital heart disease corrections were both more common and led to a higher risk of complications during the autumn and winter than in spring and summer [9]. Interestingly, a retrospective analysis including 9838 patients in Hungary revealed that cardiac interventions were more frequent during the winter months in diabetic and smoking subjects; meanwhile, during the summer months, cardiac operations predominated in older, non-diabetic and non-smoking patients [37]. Furthermore, the frequent occurrence of influenza virus epidemics during cold seasons influenced morbidity and mortality and the length of hospital stay in cardiac surgery and interventional cardiology due to the increased risk of contracting bacterial pneumonia [38,39].

An analysis of data from the transcatheter aortic valve replacement (TAVR) registry of our center (Geneva University Hospital, N = 530), confirms the higher risk profile of patients requiring TAVR intervention in winter compared to summer. The prevalence of coronary heart disease is higher in patients undergoing TAVR in winter (35.4% vs. 25.2% in summer), with an increased risk of presenting at least one post-interventional complication (37.7% vs. 22.4% in summer). As summarized in Table 1, an analysis of large databases and single-center registries indicates that postoperative mortality, major complications and ICU length of stay are higher when cardiac procedures are performed during winter or autumn, compared with spring or summer [37,38,39,40,41,42,43].

### 4.2. Circadian Variations and Cardiac Interventions

Cardiac surgery with aortic cross-clamping represents an ideal human model of ischemia–reperfusion for evaluating the impact of the circadian rhythm on the occurrence of myocardial lesions and functional recovery whenever other confounding factors are well controlled. In a prospective single-center cohort study including patients undergoing aortic valve surgery (N = 596), Montaigne et al. performed a propensity-score-matched analysis and reported a lower incidence of major adverse cardiac events (myocardial infarctions, heart failure) in the afternoon surgery group compared with the morning group [44]. In a randomized controlled trial (N = 88), these investigators confirmed that lesser myocardial injuries occurred in the afternoon surgical groups whereas acute kidney injury was not influenced by the daytime schedule of surgery [45]. Lending support to cardioprotective effects prevailing in the afternoon, a transcryptomic analysis of circadian genes on myocardial samples revealed inverse variations in the expression of the Rev-Erbα and BMAL1/ARNTL genes in patients operated on in the afternoon compared to those operated on in the morning; these genomic changes paralleled a better (vs. worse) tolerance to ischemia-reoxygenation [44].

Yet, these favorable results regarding performing surgery in the afternoon have not been replicated so far. Teams of clinicians–researchers have retrospectively examined data from a randomized study (N = 124, coronary artery bypass grafts) [46] and those from cohort studies by creating pairs of patients operated on in the morning or afternoon while adjusting for potential confounding factors using propensity score matching (Table 2) [47,48,49,50,51]. The meta-analysis of these data indicates that interventions scheduled in the morning or afternoon are associated with comparable rates of mortality (Odds Ratio [OR] 1.1, 95% confidence interval [CI] 0.85–1.42) and perioperative myocardial infarcts (OR 1.0, 95% CI 0.64–1.57) [52,53]. An analysis of other cohort studies including various types of cardiac operations also failed to demonstrate any association between the circadian schedule of surgery and the postoperative outcome [52,53,54]. Moreover, starting non-emergent cardiac cases after 3 pm has been associated with a twofold higher operative mortality after adjustment for the type of surgery, the operator and the preoperative risk, despite a shorter operative duration. Neurocognitive capacities and the physical skills of healthcare workers could be reduced over time, particularly after prolonged and complex operations.

In interventional cardiology, data from the literature indicate that the circadian rhythm is not associated with a higher/lower risk of complications occurring after TAVR and percutaneous coronary interventions (Table 2). For the TAVR procedures listed in the PARTNER trials, mortality and rehospitalizations up to 2 years post-intervention were similar in the groups operated on in the morning and the afternoon [49]. Analysis of the Geneva TAVR registry indicates a similar cardiovascular risk profile for the two groups (morning, afternoon) and confirms the absence of a difference in post-interventional clinical outcomes. Following elective percutaneous angioplasties, Fournier et al. observed a higher incidence of myocardial infarcts when elective procedures were scheduled in the afternoon (29% vs. 21% in the morning) [55].

### 4.3. Limitations of Current Scientific Knowledge

To date, although animal studies strongly support the impact of circadian rhythms on the tolerance to myocardial ischemia–reperfusion, retrospective studies have failed to report any differences in patient clinical outcomes, with respect to the time of day at which cardiac surgeries are performed. These cohort studies, comparing groups of consecutive patients operated on in the afternoon to those operated on in the morning, had numerous shortcomings and weaknesses. The lack of randomization favors significant bias, with inhomogeneities in patient groups (comorbidities, quality of care, skill level of caregivers). The potential beneficial effects of starting interventions in the afternoon could be neutralized by prolonged fasting and patient anxiety, team fatigue, a reduced availability of experienced/skilled personal and the disruption of care processes with impaired night-time quality of postoperative care. Anesthesia-related problems have been shown to increase from a low of 1% when surgeries are started at 9 a.m. to a high of 4.2% for those starting at 4 p.m. [56]. In a recent meta-analysis of 19 retrospective cohort studies focused on elective noncardiac surgery, an evening/night-time elective schedule was associated with a higher risk of mortality compared with daytime surgery; meanwhile, no difference was reported when comparing morning to afternoon surgery [57]. Furthermore, patients in need of cardiac interventions are at a higher risk of a disrupted or dampened circadian rhythm, potentially diminishing daytime variations in postoperative complications [58,59].

**Table 2:** Clinical studies reporting circadian variations in clinical and functional outcomes after cardiac surgery and cardiological procedures.

Authors	Year Country	N	Type of Surgery	Mortality	Postoperative Outcome Other Results
Yount KW et al. [60]	2015 Canada	3395 Single center	Elective heart surgery	↑ in-hospital mortality in Afternoon (5.2% vs. 3.5% Morning), OR 2.04	Similar incidence of postoperative complications
Montaigne D et al. [44]	2018 France	596 Single center	SAVR	no difference in operative mortality between Afternoon and Morning operations	↓ CV complications in the Afternoon group (9% vs. 18% Morning group); no difference in hospital LOS
Montaigne D et al. [44]	2018 France	88, RCT Single center	SAVR	no difference in mortality rate	↓ cTp-T in Afternoon; no differences in CV support, AF, hospital LOS
Montaigne D et al. [44]	2018 France	30 Single center	in vitro study SAVR	-	↑ contractility after hypoxia-reoxygenation in samples harvested in the Afternoon (vs morning) ↓ Rev-Erbalpha gene expression
Baik J et al. [52]	2019 South Korea	1690 Single center	Off-pump CABGS	No difference 1-year mortality (2.7% Morning vs. 1.5% Afternoon)	similar 30-day major CV complications (10.5% Afternoon vs. 8.9% Morning), renal dysfunction and release of cTp-T
Gotte J et al. [48]	2020 Germany	2720 Single center	SAVR isolated or with CABGS	Similar 30-day mortality (1.5% Afternoon vs. 2.7% Morning)	Similar risk of MI in afternoon vs morning groups (HR 0.88, 95% CI 0.32–2.38) and heart failure (HR 0.91, 95% CI 0.65–1.28)
Kenney PS et al. [51]	2020 Danemark	7148 Single center	SAVR isolated or with CABGS	Similar 30-day mortality (1.5% Afternoon vs. 1.5% Morning)	Similar 30-day major CV complications (3.8% Afternoon vs. 3.3% Morning), MI ((2.4% vs. 2.0%), AF (20.6% vs. 21.4%), renal failure and hospital LOS
Nemeth S et al. [47]	2021 USA	14,078 Multicenter registry	CABGS (10,863), SAVR (3215)	Similar 30-day mortality for CABGS (1.5% Afternoon vs. 1.3% Morning) and for SAVR (1.6% Afternoon vs. 1.7% Morning)	Similar risk of 30-day MI, stroke, renal failure, infections, prolonged ventilation
Fudulu DP et al. [50]	2021 UK	105,459, National registry	CABGS (78,232), SAVR (27,227)	Similar 30-day mortality (1.0% Afternoon vs. 1.0% Morning)	↑ preoperative risk factors in the Morning group (↓ LVEF, ↑ renal dysfunction, PHT and MI)
Moscarelli M et al. [46]	2021 Italy	124 Single center	CABGS, post-hoc analysis (RCT)	-	Similar postoperative release of cTp, energetic substrate in cardiac tissue samples (ATP/ADP and ATP/AMP ratios)
Michaud M et al. [54]	2012–2018-Canada	538 Single center	SAVR with/without CABGS	Similar 30-day mortality (5.2% Afternoon vs. 2.0% Morning)	Similar postoperative cTp MI, AKI, stroke and hospital LOS
Immohr MB et al. [53]	2024-Germany	235 Single center	Heart transplantation	No difference in 30-day mortality (9.2% Afternoon vs. 11.4% Morning)	Similar rates of postoperative AKI, infections, and acute graft rejection
Fournier S et al. [55]	2014 Switzerland	1021 Single center	PCI	N.R.	↑ Post-PCI MI in the Afternoon
Vincent F et al. [49]	2014 International	5586 Multicenter	TAVR (4457) SAVR (1129)	No difference in 30-day mortality after TAVR (8.6% Afternoon vs. 8.1% Morning) and after SAVR (8.6% Afternoon vs. 9.1% Morning)	Similar rate of AKI, new pacemaker and major bleeding in the Afternoon and Morning groups after TAVR and SAVR Similar functional recovery after TAVR and SAVR

AKI, acute kidney injury; CABGS, coronary artery bypass graft surgery; CI, confidence interval; ICU, intensive care unit; LOS, length of stay; MI, myocardial infarct; OR, odds ratio; PCI, percutaneous coronary interventions; PHT, pulmonary hypertension; SAVR, surgical aortic valve surgery; TAVR, transcatheter aortic valve replacement; cTp, cardiac troponin; ↓ and ↑, decrease and increase.

## 5. Conclusions

Although the scientific evidence for the impact of the circadian rhythm on myocardial ischemia–reperfusion tolerance is solid, the retrospective analysis of large databases shows that the “timing” of cardiovascular interventions, namely in the morning or the afternoon, is poorly related to the occurrence of major postoperative complications. Healthcare worker-associated issues (e.g., expertise, vigilance, neurocognitive and physical capacities), the availability of resources, clinical care pathways and the burden of pathological processes all influence patients’ outcomes. The retrospective design of the aforementioned studies precluded the analysis of these important confounding factors.

Therefore, we need well-designed, prospective studies using strong methodologies that take into account daily time slots, patients’ comorbidities, standardized perioperative anesthetic and surgical management and chronotherapy to provide a thorough understanding and definite answers regarding the impact of the timing of major interventions on postoperative outcomes.

## Figures and Tables

**Table 1:** Clinical studies reporting seasonal variations in major complications after cardiac surgery.

Authors	Year-Country	N	Type of Surgery	Mortality	Postoperative Outcome Other Results
Shuhaider JH et al. [40]	2008-UK	16,290 Single center	isolated or combined CABGS	↑ in-hospital mortality after isolated CABGS (OR 1.29, 95%CI 1.01–1.63) in winter	Prolonged ICU stay after isolated CABGS in winter
Mori et al. [39]	2020-USA	448,709 National database	CABGS, SAVR	Trend for increased risk of operative mortality during severe influenza epidemics (OR 1.03, 95%CI 0.96–1.10)	N.R.
Martin TJ et al. [38]	2020-USA	516,698 National database	CABGS	↑ Risk of postoperative pneumonia (OR 1.15, 95%CI 1.07–3.12) and viral infection (OR 4.1, 95%CI 2.0–7.9) during winter	Preoperative vaccination against seasonal influenza, Hemophilus influenzae, and S. pneumoniae improve postoperative outcome
Luo Z-L et al. [41]	2021-China	404 Single center	Aortic dissection	↑ In-hospital mortality in autumn (OR 4.0, 95%CI 1.0–17.3) and patients with CAD (OR 9.0, 95%CI 2.0–29.6)	Prolonged ICU stay in patients operated on during autumn (OR 6.0, 95% CI 2.7–7.9)
Petak F et al. [37]	2022-Hungary	9838 Single center	CABGS, SAVR	N.R.	↑ younger patients with diabetes and smokers operated in winter No seasonality variation regarding type of surgery ↑ BP and ↑ plasma triglyceride levels in winter
Lin Q et al. [42]	2023-China	485 single center	Aortic dissection	Similar in-hospital mortality during winter (12.3%), spring (10.4%), summer (9.6%) and autumn (9.8%)	Prolonged hospital LOS during winter (median 20 days, [IQR] 2–31) vs. summer (17, IQR 4–24)
Swets MC et al. [43]	2023-Netherlands	42,277 National database	CABGS	↑ in-hospital mortality (OR 1.67, 95% CI 1.14–2.46) during autumn and winter (influenza- like illness)	Worse outcome due to ILI epidemics, in October (vs. April)

CABGS, coronary artery bypass graft surgery; CI, confidence interval; ICU, intensive care unit; ILI, influenza-like illness; IQR, interquartile range; LOS, length of stay; N.R., not reported; OR, odds ratio; SAVR, surgical aortic valve surgery; TAVR, transcatheter aortic valve replacement; ↑, increase.
